# Lived experience-centred word clouds may improve research uncertainty gathering in priority setting partnerships

**DOI:** 10.1186/s12874-023-01897-6

**Published:** 2023-04-22

**Authors:** Oliver D. Mowforth, Lance Burn, Danyal Z. Khan, Xiaoyu Yang, Sybil R. L. Stacpoole, Toto Gronlund, Lindsay Tetreault, Sukhvinder Kalsi-Ryan, Michelle L. Starkey, Iwan Sadler, Ellen Sarewitz, Delphine Houlton, Julia Carter, Paige Howard, Vafa Rahimi-Movaghar, James D. Guest, Bizhan Aarabi, Brian K. Kwon, Shekar N. Kurpad, James Harrop, Jefferson R. Wilson, Robert Grossman, Emma K. Smith, Angus McNair, Michael G. Fehlings, Mark R. N. Kotter, Benjamin M. Davies

**Affiliations:** 1 Division of Neurosurgery, Department of Clinical Neurosciences, University of Cambridge, Cambridge, UK; 2 Department of Clinical Neurosciences, North West Anglia NHS Foundation Trust and Cambridge University Hospitals NHS Foundation Trust, Cambridge, UK; 3 James Lind Alliance, National Institute for Health Research, Southampton, UK; 4 Division of Neurosurgery, University of Toronto, Toronto, ON Canada; 5 Toronto Rehabilitation Institute-LC, University Health Network, Toronto, ON Canada; 6 Myelopathy.Org, (Registered Charity England and Wales, No 1178673), Cambridge, UK; 7 The Goffin Consultancy, Goffin Consultancy Ltd, Riding House, Stelling Minnis, Bossingham Road, Canterbury, CT4 6AZ UK; 8 US Person with DCM Representative – CSU, Bakersfield, CA USA; 9 Academic Department of Neurological Surgery, Sina Trauma and Surgery Research Center, Tehran University of Medical Sciences, Tehran, Iran; 10 Department of Neurological Surgery, Miller School of Medicine, University of Miami, Miami, FL USA; 11 Division of Neurosurgery, Shock Trauma, University of Maryland, Baltimore, MD USA; 12 Division of Spine Surgery, Vancouver General Hospital, University of British Columbia, Vancouver, BC Canada; 13 Department of Neurosurgery, Medical College of Wisconsin, Milwaukee, WI USA; 14 Division of Neurosurgery, Thomas Jefferson University Hospital, Philadelphia, PA USA; 15 Division of Neurosurgery, Houston Methodist Hospital, Houston, TX USA; 16 School of General Practice, NHS Health Education East of England, London, UK; 17 Center for Surgical Research, Bristol Medical School: Population Health Sciences, University of Bristol, Bristol, UK; 18 Wellcome Trust & MRC Cambridge Stem Cell Institute, Cambridge, UK

**Keywords:** Cervical, Myelopathy, Word cloud, Ossification of posterior longitudinal ligament, Spondylosis, Disc herniation, Cervical stenosis, Outcome, Research priorities, Consensus, Audit, Surveillance, Common data elements

## Abstract

**Introduction:**

AO Spine RECODE-DCM was a multi-stakeholder priority setting partnership (PSP) to define the top ten research priorities for degenerative cervical myelopathy (DCM). Priorities were generated and iteratively refined using a series of surveys administered to surgeons, other healthcare professionals (oHCP) and people with DCM (PwDCM). The aim of this work was to utilise word clouds to enable the perspectives of people with the condition to be heard earlier in the PSP process than is traditionally the case. The objective was to evaluate the added value of word clouds in the process of defining research uncertainties in National Institute for Health Research (NIHR) James Lind Alliance (JLA) Priority Setting Partnerships.

**Methods:**

Patient-generated word clouds were created for the four survey subsections of the AO Spine RECODE-DCM PSP: diagnosis, treatment, long-term management and other issues. These were then evaluated as a nested methodological study. Word-clouds were created and iteratively refined by an online support group of people with DCM, before being curated by the RECODE-DCM management committee and expert healthcare professional representatives. The final word clouds were embedded within the surveys administered at random to 50% of participants. DCM research uncertainties suggested by participants were compared pre- and post-word cloud presentation.

**Results:**

A total of 215 (50.9%) participants were randomised to the word cloud stream, including 118 (55%) spinal surgeons, 52 (24%) PwDCM and 45 (21%) oHCP. Participants submitted 434 additional uncertainties after word cloud review: word count was lower and more uniform across each survey subsections compared to pre-word cloud uncertainties. Twenty-three (32%) of the final 74 PSP summary questions did not have a post-word cloud contribution and no summary question was formed exclusively on post-word cloud uncertainties. There were differences in mapping of pre- and post-word cloud uncertainties to summary questions, with greater mapping of post-word cloud uncertainties to the number 1 research question priority: raising awareness. Five of the final summary questions were more likely to map to the research uncertainties suggested by participants after having reviewed the word clouds.

**Conclusions:**

Word clouds may increase the perspective of underrepresented stakeholders in the research question gathering stage of priority setting partnerships. This may help steer the process towards research questions that are of highest priority for people with the condition.

**Supplementary Information:**

The online version contains supplementary material available at 10.1186/s12874-023-01897-6.

## Introduction

Degenerative cervical myelopathy (DCM) is the most common cause of spinal cord impairment worldwide [1]. It arises when degenerative pathology in the cervical spine precipitates a subacute compression injury to the cervical spinal cord. Despite the best current management [2], many people with DCM are left with life-changing disabilities [3] and amongst the worst quality of life of all chronic diseases [4].

AO Spine RECODE-DCM (REsearch objectives and Common Data Elements for Degenerative Cervical Myelopathy) was a multi-stakeholder, international consensus process aiming to accelerate DCM research through recommendations that improve research efficiency [5]. It combined several consensus initiatives, including establishing the top 10 DCM research uncertainties [6–19].

The process of establishing research priorities is supported by organisations such as the National Institute for Health Research (NIHR) James Lind Alliance (JLA) [20]. The JLA define research uncertainties as (1) areas with no up-to-date, reliable systematic reviews of research evidence addressing the uncertainty, or (2) up-to-date systemic reviews of research evidence showing that uncertainties exist [21]. JLA methodology starts with an information gathering process, by seeking research suggestions from patients, family, caregivers, and healthcare professionals. Commonly this is conducted using web-based surveys, with survey design tailored to the specific condition under consideration. For AO Spine RECODE-DCM, survey sub-sections included diagnosis, treatment, long-term management, and other issues [5]. The process is designed to build consensus and encompasses several rounds of surveys. After each round participants are presented with an anonymised summary of all responses and can amend their individual responses [22].

A major driver of research inefficiency is the underrepresentation of end-users, including people living with degenerative cervical myelopathy (PwDCM), within research design [21, 22]. Involvement of end users, such as via surveys, focus groups and online crowdsourcing [23], has been shown to be essential for research results to be of value [24–26] While this is recognized in PSP methodology by participation of end users in the surveys and as steering committee members, the perspectives of the whole end-user population is often not clear to the wider group until the final consensus meeting. Moreover, studies of JLA PSP methodology have identified that current PSP surveys may underrepresent end-user perceptions in favour of the views of healthcare professionals [27]. In particular, end users with physical, language and communication disabilities may face the greatest challenges in accessibility, participation and engagement within current PSPs [28, 29]. We therefore hypothesized that the end user perspective could be integrated earlier in the process by including end-user generated word clouds in the information gathering phase to help stimulate research uncertainty suggestions from other stakeholders.

We also hypothesised that word clouds would help mitigate against a unique challenge for the DCM PSP, that a diversity of healthcare professionals is involved in DCM care, but generally, the practice of each healthcare professional group is confined to a limited stage of the disease, such as initial diagnosis [30, 31]. This narrow focus of each practitioner’s role has been proposed to limit clinical research creativity [32].

Word clouds are a tool which enable qualitative data to be displayed, with the relative importance or frequency of each idea is proportional to the size of the word in the cloud. In the medical literature, word clouds have mainly been used to report qualitative patient interview data [33], although further afield they have been utilised to stimulate creativity [26, 27, 34].

The methodology for the generation of the word clouds used in the AO Spine RECODE-DCM PSP has been described in detail elsewhere [35]. Here we evaluate their impact on the responses in the PSP process. The aim was to provide insight into the role and value of word clouds in stimulating a creative, end-user focused research agenda in PSPs. We hypothesised that the incorporation of lived-experience-centred word clouds may more effectively identify research uncertainties and priorities.

## Methods

A detailed description of the methodology used to design and create the AO Spine RECODE-DCM word clouds has been published [35].

In brief, word clouds were generated in collaboration with Myelopathy Support, an arm of Myelopathy.org, which is an international DCM charity. Myelopathy Support is an online community hosted on Facebook (Meta, California, USA). The group is closed, and access is moderated by Myelopathy.org volunteers. Individuals wishing to join the group are required to confirm they have myelopathy and will adhere to community guidelines. Prior experience has demonstrated that demographics and disease characteristics of this group, aside from a female gender predominance, are broadly representative of DCM clinical trials [36–40]. Ethical approval was not required for the involvement of the Myelopathy Support members as their role was to help to develop the word clouds for the survey and they were not research participants [41]. The lead moderator of Myelopathy Support (IS) is a member of the AO Spine RECODE-DCM management group and steering committee.

Over a 2-week period, 4 posts were pinned to the top of the Myelopathy Support group: (1) “What words do you associate with the diagnosis phase of myelopathy?” (2) “What words do you associate with the management phase of myelopathy?” (3) “What words do you associate with the long-term care/living with myelopathy?” (4) “Any other relevant words?”. The posts were accompanied by covering information outlining the background to the exercise, piloted and approved by IS. Group members were encouraged to add their suggestions as comments on the posts and to vote for the words with which they agreed. No data was collected on individual participants. There was no limit on how many times a group member could contribute; each member could vote for each word only once.

Following the 2-week period, the submitted words and their respective votes were reviewed by the AO Spine RECODE-DCM Management Group [ODM, ES, DK, IS, BMD, OH] and duplicated words were removed. Words considered to be out of scope were removed and words felt to be better reflected in a different section were moved. Through consultation with representatives of the other healthcare professional (oHCP) stakeholder group (a general practitioner (EKS) and a neurologist (SRLS) and discussion amongst the management group, some additional suggestions were added. The aim of this was to include the perspective of another under-represented stake-holder group [42] ensuring the process remained patient-centred by presenting suggestions back to the Myelopathy Support group for a further round of voting. Full details on the process of creating the word clouds have been published elsewhere [35].

**Fig. 1:**
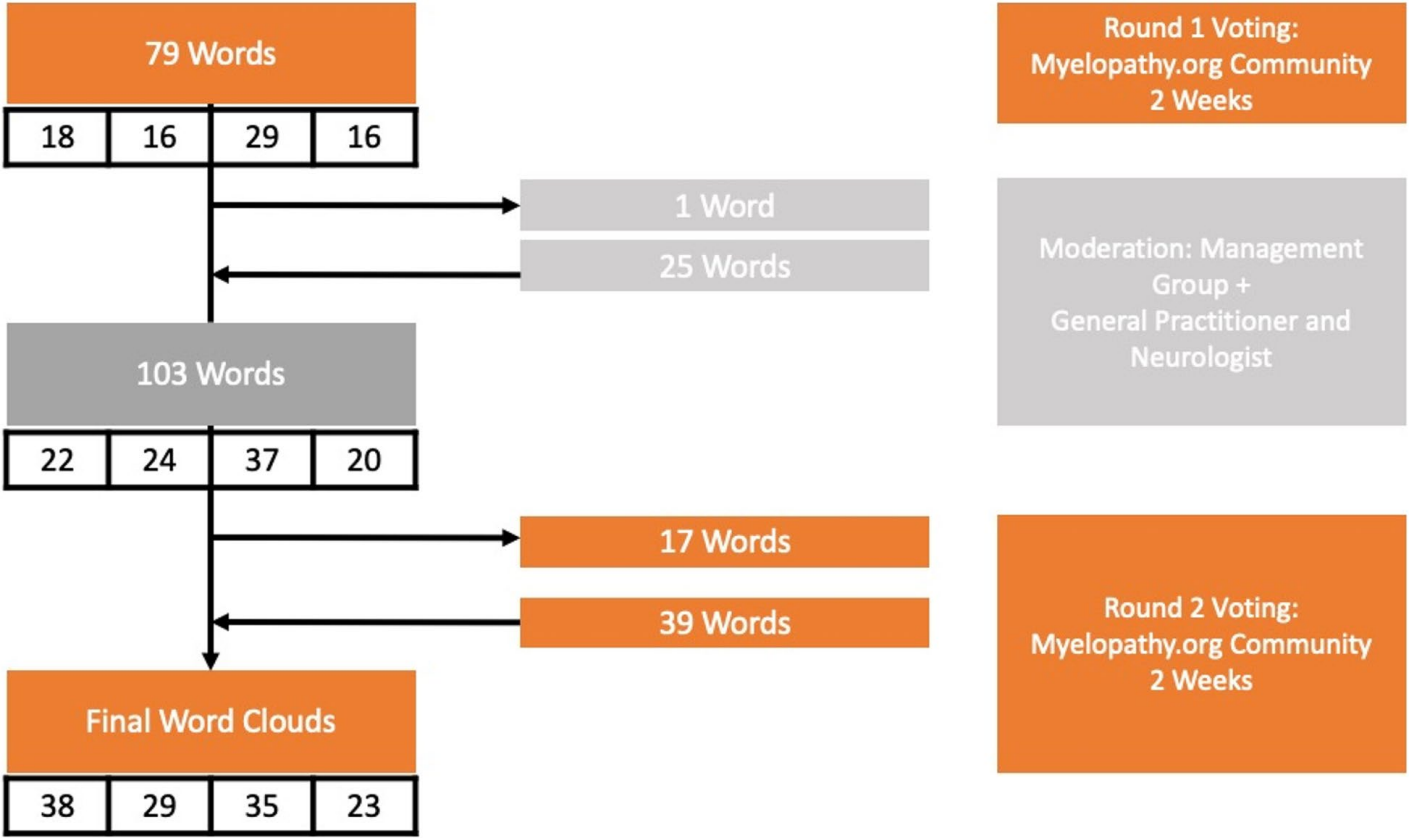
Word Cloud Development. A 3-stage process was used to collect unique words relating to DCM and survey their popularity to create word clouds. Words were initially submitted by Myelopathy Support, a community of PwDCM and their supporters. These were then processed by the AO Spine RECODE-DCM Management Group and supplemented with the perspectives of a general practitioner and a neurologist; arrows denote added and removed words. The final list of words was re-reviewed by the Myelopathy Support community. Voting was conducted to capture the popularity of each word and further suggestions were also put forward. The four-column tables represent the number of words per subsection, ordered from left to right as: diagnosis, treatment, long-term management and other

The final lists were re-posted to the group as a series of polls organised to allow members to review the list and vote for the words with which they most agreed. Members could continue to submit additional suggestions, which were then available to be polled. Again, members could not vote more than once for each word. This second-round exercise ran for a further 2 weeks. Final suggestions and polling were reviewed by the management group, with removal of duplicates and out of scope suggestions. A flow chart of the processes used is shown in Fig. 1.

**Fig. 2:**
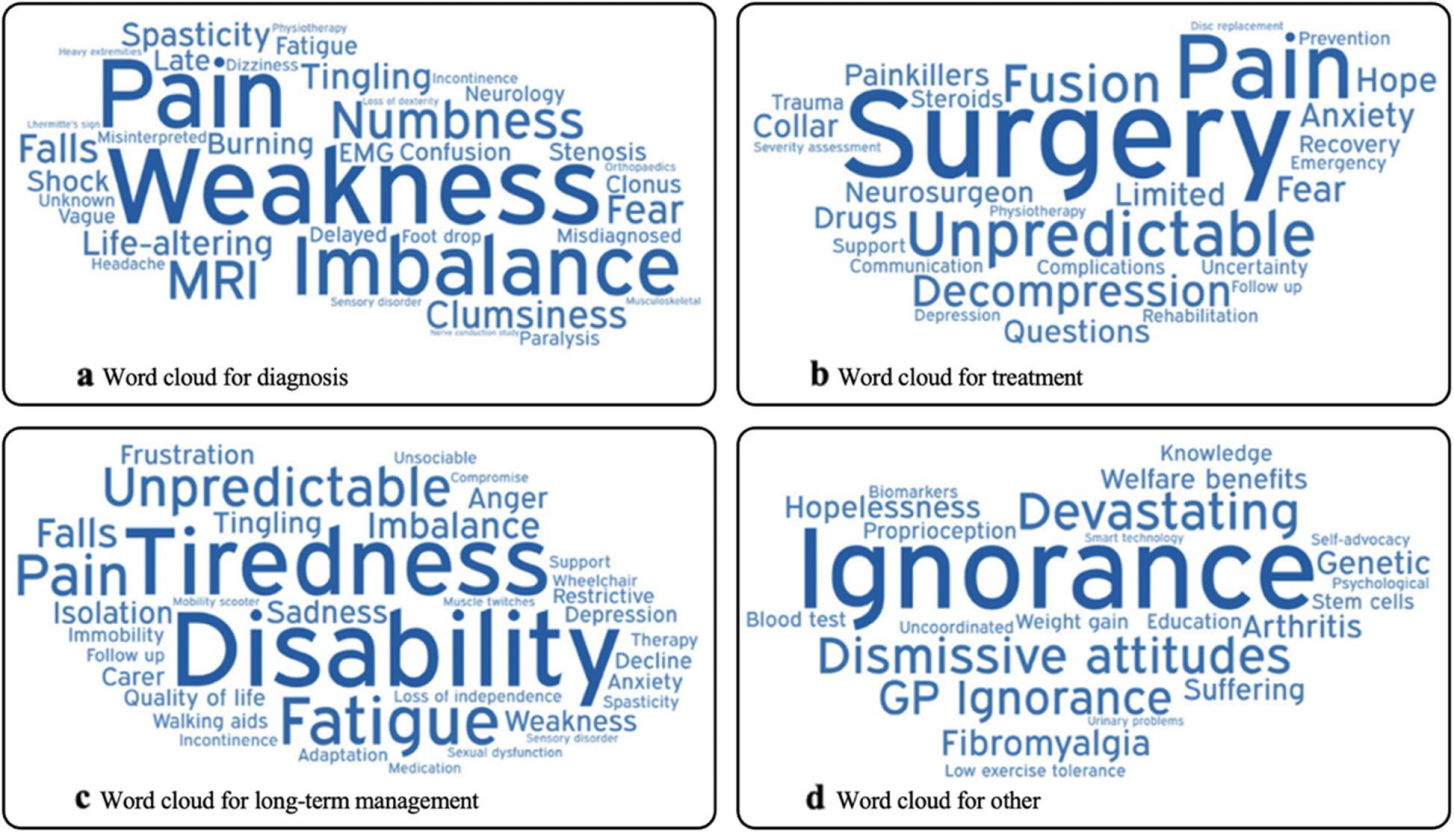
Word clouds generated from the Myelopathy Support group polling. Words were associated with (**a**) DCM diagnosis, (**b**) treatment, **(c)** long-term management and (**d**) other. The size of the word is proportional to the number of votes it received during polling

Word clouds were generated using WordArt.com (California, USA). The size of the word was proportional to the number of votes it received (Fig. 2). Full polling results have been reported elsewhere [35]. In summary, the maximum number of votes received by a word was 49. Words without a vote were not included in the clouds. The first iteration of the final output was used, without modification.

### Evaluation of word cloud utility in generating PSP summary questions

**Fig. 3:**
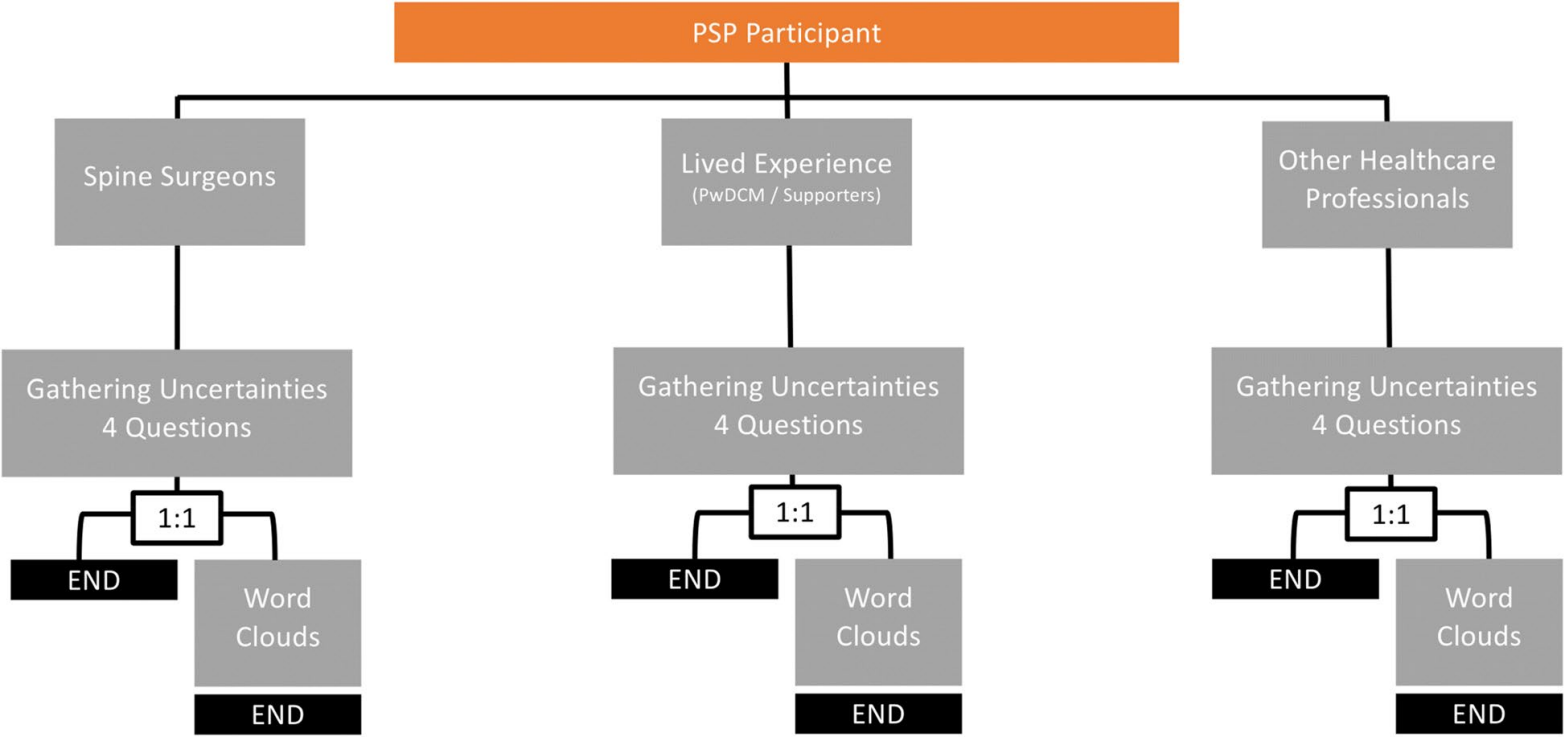
Incorporation of word clouds in the PSP. Participants allocated to the AO Spine RECODE-DCM PSP were categorised by stakeholder group (spinal surgeons, PwDCM and their supporters, other healthcare professionals). Participants within each stakeholder group were randomly allocated to word cloud or no word cloud streams using a computerised 1:1 block randomisation protocol. Participants in the word cloud stream were presented with the word clouds after the standard survey seen by the non-word cloud group and then given the opportunity to submit further uncertainties

AO Spine RECODE-DCM contained several parallel consensus processes and included three main stakeholder groups: (1) spinal surgeons, (2) PwDCM and their supporters, (3) other healthcare professionals. Consent was obtained from all participants. For the PSP arm of RECODE-DCM, participants were randomised by stakeholder group using a 1:1 computerised block randomisation protocol, to see the traditional PSP survey alone, or the traditional PSP survey followed by the word clouds (Fig. 3). This approach was taken to ensure adherence to the standard JLA methodology, whilst simultaneously allowing evaluation of the word clouds. Participants were able to move freely within the survey up until the point they submitted their responses. Consequently, it was theoretically possible for participants in the word cloud stream to edit their pre-word cloud responses having seen the word clouds. Whilst this cannot be tracked by the survey platform, by retaining an arm who do not have access to the word clouds, suspicious activity could be evaluated and was not noted. The wider methodology and governance of RECODE-DCM has been reported in detail elsewhere [7].

### Data analysis

All suggested research uncertainties were then processed as is standard for a JLA PSP [8]. In short, uncertainties were reviewed by an information specialist (LT), who was specifically recruited to AO Spine RECODE-DCM to manage the data. Her role was to review the responses to the survey, organise and categorise the submitted uncertainties into themes, remove out of scope uncertainties, generate clearly formatted summary questions, check existing literature to verify that each summary question was a true research uncertainty and present summary questions to the steering committee for discussion. As per JLA protocol, an audit trail was maintained from submitted uncertainties to the final list of top ten research priorities and this process was overseen by the steering committee. This methodology has been reported in detail elsewhere [8].

In addition, submitted research uncertainties were coded by stream (word cloud vs. no word cloud). For those within the word cloud stream, the uncertainties were coded as arising either before or after seeing the word clouds. The number of suggested research uncertainties that were grouped under a particular summary question in the final analysis were recorded for each stage of the survey (without word clouds, before/after word clouds). To evaluate the impact of word clouds on uncertainties-gathering, the following results were compared before and after reviewing the word-clouds:

1. Median word count of suggested uncertainties
2. Mapping of uncertainties to summary questions
3. Number of submitted uncertainties informing summary questions

### Statistical analysis

Data analysis was conducted in Microsoft Excel (Microsoft 365, Washington, USA). As an exploratory analysis, data is presented with summary statistics only.

## Results

The AO Spine RECODE-DCM PSP recruited a total of 422 participants: 215 (50.9%) participants were randomised to the word cloud stream. Participants in the word cloud stream consisted of 118 (55%) spinal surgeons, 52 (24%) PwDCM and 45 (21%) oHCP. A total of 157 (73%) participants in the word cloud stream submitted additional uncertainties following word cloud presentation, including 86 (55%) spinal surgeons, 28 (18%) oHCP and 43 (27%) PwDCM. In total, participants submitted 434 additional research uncertainties after having reviewed the word clouds.

**Fig. 4:**
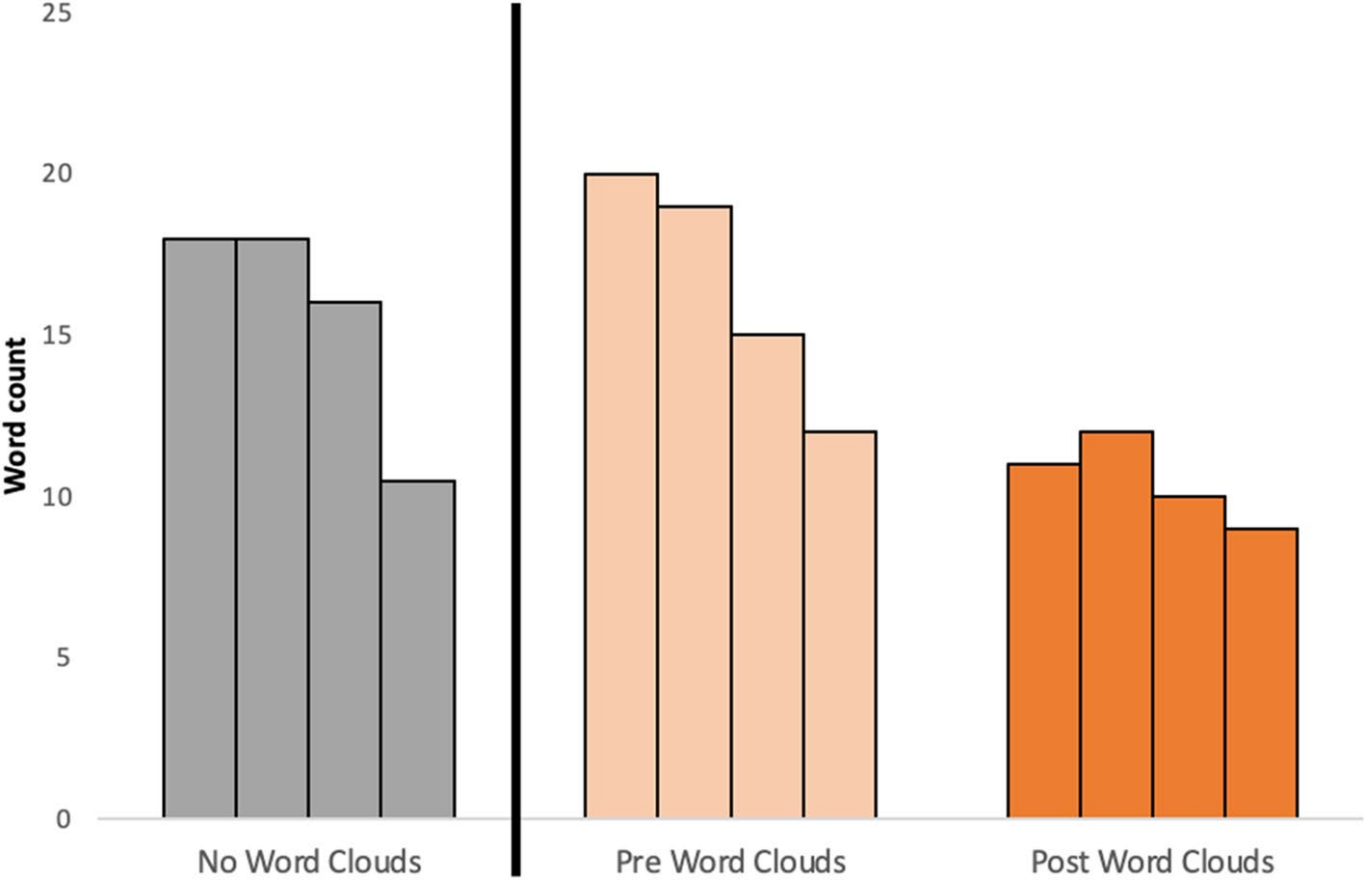
Median number of words submitted per survey subsection (diagnosis, treatment, long-term management and other). Each group of bars represents subsections at different stages: without word clouds (grey), before word clouds (pale orange) and after word clouds (dark orange). Ordered from left to right, the bars represent diagnosis, treatment, long-term management and other**.** The vertical black line separates the two streams. The number and distribution of submissions across subsections was similar for no words clouds and pre-word clouds. The number of words submitted post word clouds was fewer and consistent across each subsection

The average word count of uncertainties per survey subsection (diagnosis, treatment, long-term management and other) were comparable between the non-word cloud stream and the word-cloud stream before word cloud presentation. However, after word cloud review word count of uncertainties was lower and more uniform across each subsection (Fig. 4).

**Fig. 5:**
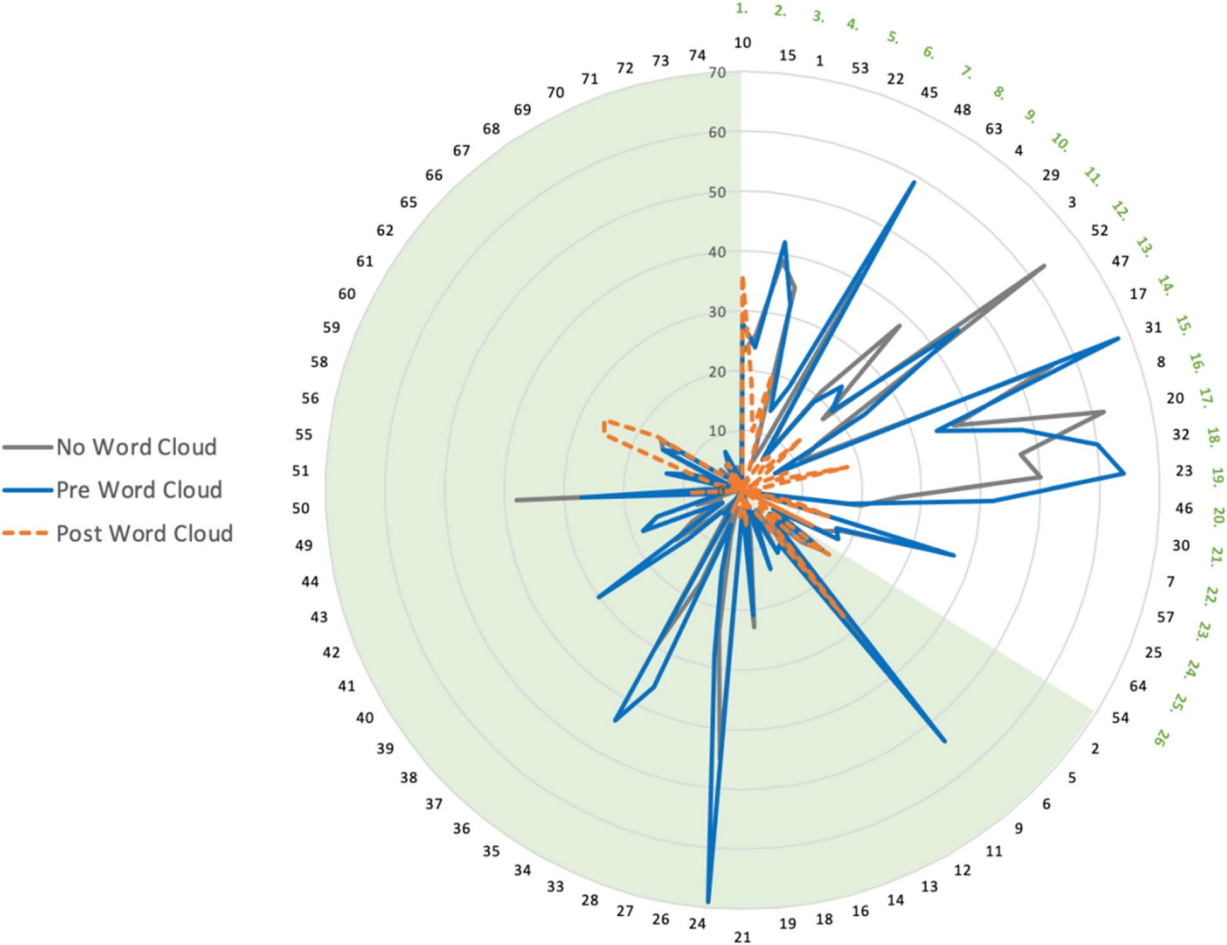
Radar plot of research uncertainties and how they map to the final 74 summary questions (black numbers) and top 26 priorities (green numbers), from uncertainties without (grey) or pre-word cloud (blue) compared to post-word cloud (orange). Post-word cloud uncertainties mapped to different research uncertainties than pre-word cloud uncertainties, including the number 1 research priority: raising awareness

Of the 74 summary questions generated, 23 (32%) summary questions did not have a post-word-cloud contribution, and no summary question was formed based purely on an uncertainty generated after review of the word clouds. However, of the 434 research uncertainties made following word cloud review, only 108 (24.9%) were already represented in a participant’s submission prior to reviewing the word clouds and these uncertainties mapped to summary questions differently compared to those submitted prior to review of the word clouds (Fig. 5).

In addition, 5 summary questions appeared more likely to map to a participant’s research uncertainties following word cloud review. Two of these summary questions were subsequently ranked in the top 26 priorities, including the top research priority: raising awareness (Table 1).

**Table 1:** Summary questions to which more uncertainties mapped after word cloud review. Numbers represent the number of responses

Summary Question	Ranking	Without Word Cloud	Pre Word Cloud	Post Word Cloud
What strategies can increase awareness and understanding of DCM amongst healthcare professionals and the public? Can these strategies help to improve timely diagnosis and management?	1	23	28	36
Can CSF or serum biomarkers be identified to support early diagnosis of DCM and/or predict treatment outcomes?	22	2	2	9
What is the role of electrophysiology in the assessment and diagnosis of DCM?	N/A	13	6	12
What is the impact of DCM on mental health? How can patients be best supported from this perspective?	N/A	6	3	25
What is the impact of DCM, and its specific complications, on long-term quality of life?	N/A	15	15	26

## Discussion

The inclusion of PwDCM-generated word clouds in a JLA PSP enabled stakeholders to generate more research uncertainties. Whilst this did not lead to the formation of additional research questions, research uncertainties submitted in response to the word clouds were more likely to map to the number one research priority: raising awareness.

The JLA, following some initial iterations, has continued to use a single and proven methodology [43]. The incorporation of word clouds in AO Spine RECODE-DCM was therefore the first example of a prospective, nested methodological study for the JLA. We have shown that such methodology can capture and highlight inputs from under-represented stakeholder groups like PwDCM. Word clouds provided other stakeholder groups a view on the interaction of PwDCM with healthcare services, which can be effectively incorporated into a protocol to influence research uncertainty identification.

Unlike many other consensus processes [24–26, 44, 45] the JLA does not incorporate shared feedback until the final consensus meeting [43]. The consequence of this is that input from certain stakeholder groups, such as patients, is not represented until the final stages of a study. In AO Spine RECODE-DCM, our ambition for the word clouds was to ensure that the perspectives of under-represented stakeholders were available for all, especially during the early information gathering phase of the process.

As only one research uncertainty is required to generate a research question, and uncertainties based on the word clouds alone did not generate a unique research question, it may appear that word clouds were unnecessary and lead to inefficiency. However, the fact that word clouds stimulated individual participants to generate new ideas reflects a positive impact on creativity. Furthermore, the identification of themes for summary questions will invariably be partly informed by the frequency of their representation [46]. Finally, it was of note that word cloud submissions were more likely to map to the number one research priority.

It is too early to conclude definitively whether word clouds had a positive impact, but these are all promising indications. Further research is required, but it is of note that the JLA has now decided to conduct a consultation looking at a role for developing a research methodology stream including how different research methodologies could affect consensus-building. This is particularly welcome in the context of an expanding literature demonstrating the challenges of end-user participation in the traditional PSP [23, 27]. In particular, strategies to promote participation of people with disabilities are most acutely needed [29, 47].

### Limitations

The present study represents an early-phase methodological innovation and is perhaps best conceptualised as a feasibility study. As such, lessons can be learnt from our experience for future studies. Firstly, a more granular analysis of the impact of word clouds on each of the 3 participants groups would be of future value. Secondly, future work should define the most appropriate outcome measures to assess the impact of word clouds and other methodological innovations in PSPs, particularly given the qualitative nature of this field, and the challenge of drawing statements of causation. It may well be that end-user perceptions of accessibility and inclusion are the most appropriate metrics, instead of more quantitative measures such as word count.

### Future implications

One of the challenges for implementation strategies in healthcare is the provision of early feedback [29, 48]. For example, a commonly used metric to evaluate strategies to accelerate evidence transfer into practice is its adoption within clinical guidelines. This will, at best, be measured in years [49]. The simple success here of feeding back current perspectives on end-user perspective using word clouds may present additional opportunities for implementation science, including monitoring the impact of research prioritisation and evaluating its need for an update.

Word clouds may also provide a practical solution for gathering perspectives in PSP projects with an end-user population that faces challenges engaging with traditional JLA methodology. For example, in a study aiming to make involvement in research more inclusive for people with complex speech and motor disorders, participants reported that, researchers and funders need to be more realistic about time, funding and accessibility to facilities to enable people with disabilities to participate [50]. Word clouds may improve participation of such groups through ease of online accessibly. Other protocols have already proven to be successful in promoting end-user involvement both online [47] and in person [27, 29]. Our approach seeks to develop this accessibility further given that word clouds: (1) do not require advanced language skills such as grammar or syntax, (2) do not require specialist healthcare professional training or input, (3) may be flexibly accessed online and respect fatigue demands of certain end-user groups, and (4) reduce in-person contact, which may be particularly pertinent in light of the recent COVID-19 pandemic.

## Conclusion

The inclusion of word clouds in a JLA PSP increased the number of research uncertainties suggested, including the frequency of uncertainties which aligned with prioritised research questions. In AO Spine RECODE-DCM, this included the theme of raising awareness, which was the number research priority.

## Supplementary Information

**Additional file 1.**

## Data Availability

The datasets used and/or analysed during the current study are available from the corresponding author on reasonable request.
